# Dietary modulation of human milk bioactives is associated with maternal FUT2 secretor phenotype: an exploratory analysis of carotenoids and polyphenol metabolites

**DOI:** 10.3389/fnut.2024.1463969

**Published:** 2024-10-09

**Authors:** Chelsey Fiecke, Meghan Crimmins, Ahsan Hameed, Clark Sims, D. Keith Williams, Lars Bode, Audrey Martinez, Aline Andres, Mario G. Ferruzzi

**Affiliations:** ^1^Arkansas Children’s Nutrition Center and Department of Pediatrics, University of Arkansas for Medical Sciences, Little Rock, AR, United States; ^2^Department of Pediatrics, Larsson-Rosenquist Foundation Mother-Milk-Infant Center of Research Excellence (MOMI CORE), and the Human Milk Institute (HMI), University of California, San Diego, La Jolla, CA, United States

**Keywords:** human milk, human milk oligosaccharides, carotenoids, polyphenols, obesity, Mediterranean meal plan

## Abstract

**Introduction:**

Maternal diet modifies profiles of human milk oligosaccharides (HMOs), carotenoids, and polyphenols in human milk (HM). However, substantial variability in profiles exists between women, highlighting the complexity of non-dietary factors modulating these profiles. The objective of this study was to carry out a secondary analysis exploring the effect of maternal diet on HM carotenoids and polyphenols and relationships between dietary modulation of HM bioactives (carotenoids, polyphenols, and oligosaccharides) and maternal α1,2-fucosyltransferase 2 (FUT2) secretor phenotype.

**Methods:**

In this pilot study, 16 exclusively breastfeeding women with obesity were enrolled between 4 and 5 months postpartum. The women were provided a 4-week meal plan consistent with the 2020 Dietary Guidelines for Americans (DGA). HM was collected for 24 h at baseline and post-intervention. Maternal FUT2 secretor phenotype was determined by 2′-fucosyllactose concentration in HM (non-secretor: < 100 nmol/ml; secretor: ≥100 nmol/ml). Concentrations of carotenoids and HMOs were determined by LC and polyphenol metabolites by UPLC–MS/MS.

**Results:**

Thirteen women completed the study (6 secretors, 7 non-secretors). The change in HM concentrations of the HMOs lacto-N-tetraose (LNT, *p* = 0.007), lacto-N-fucopentaose II (LNFP II, *p* = 0.02), difucosyllacto-N-tetraose (DFLNT, *p* = 0.003), and disialyllacto-N-tetraose (DSLNT, *p* = 0.003) and polyphenol metabolites 4-hydroxybenzoic acid (4-HBA, *p* = 0.08) and ferulic acid (*p* = 0.02) over the intervention time frame was differentially associated with maternal secretor status. 4-HBA and ferulic acid positively correlated with HMOs LNT and DSLNT (*r_rm_* = 0.82–0.90, *p* = 0.03–0.06) for secretors but not for non-secretors. Only secretors demonstrated a negative correlation between 4-HBA and DFLNT (*r_rm_* = −0.94, *p* = 0.001).

**Discussion:**

The influence of maternal diet on composition of HMOs and polyphenol metabolites in HM differs based on maternal secretor status. Consideration of non-dietary factors is needed to evaluate differences in response of HM bioactives to dietary modulation.

## 1 Introduction

Human milk (HM) is recognized as the optimal source of infant nutrition by the World Health Organization (WHO) (1) and the American Academy of Pediatrics (AAP) (2). Human milk has a unique composition of various bioactive components, including HM oligosaccharides (HMOs) (3, 4), carotenoids (5, 6), and polyphenols (6–8). Growing clinical evidence suggests that intake of HMOs (9–12), carotenoids (13–16), and polyphenols (17, 18) by breastfed infants supports gut and potentially cognitive development. Since carotenoids and polyphenols are not produced endogenously, they must be supplied by maternal dietary intake, which has been identified as a key factor driving concentrations of both bioactives in HM (7, 17, 19–23). Although HMOs are not derived directly from dietary intake, maternal diet has also been demonstrated to modify or associate with profiles of HMOs (24–27).

While the primary driver of carotenoid profiles in HM is maternal diet (19–22), there are multiple drivers of the variability observed in HMOs (25, 28). Other than maternal diet, known drivers of HMO variability include maternal genetics, such as α1,2-fucosyltransferase 2 (FUT2) secretor status (29, 30), and lactation duration (30–32). Variability is also observed in HM polyphenol profiles, despite similar maternal polyphenol intake (17, 18, 33), highlighting the fact that, like HMOs, profiles could be modified by non-dietary factors. Recent observational studies have suggested that maternal polyphenol intake is associated with HM microbiome composition (24) and HMO profiles (25). Associations between polyphenol intake and HMO profiles may depend on maternal secretor status (25), which could be due to differences in HM microbiome composition associated with maternal secretor status (34). Because it is well-established that dietary polyphenols undergo extensive gut microbial metabolism (35, 36), it is possible that a relationship between maternal polyphenol intake, secretor status, and composition of HM microbiota, polyphenols, and oligosaccharides exists. Integrated approaches are needed to improve our understanding of factors driving composition of HM bioactives that are diet-derived or diet-driven, such as oligosaccharides and polyphenols in HM. Thus far, no human studies have explored the role of maternal diet on diet-driven HM bioactive components when considering maternal secretor status as a factor. The present study was a secondary analysis of a within-subject pilot intervention study that was designed to determine if adherence to a healthy diet during lactation would influence the macronutrient and bioactive (hormone, HMO, cytokine) composition of HM in women with obesity, as described previously (26). There were two objectives of this secondary analysis: (1) explore the effect of maternal diet on carotenoids and polyphenols in HM and (2) carry out an exploratory analysis of relationships between dietary modulation of HM bioactives (HMOs, carotenoids, polyphenols) and FUT2 secretor phenotype.

## 2 Materials and methods

### 2.1 Chemicals and reagents

Ammonium acetate and LC–MS grade acetonitrile, methanol, formic acid, and water were purchased from Fisher Scientific (Waltham, MA, USA) and Alfa Aesar (Ward Hill, MA, USA). Potassium hydroxide (KOH) and HPLC-grade ethyl acetate were purchased from Sigma-Aldrich (St. Louis, MO, USA). ACS-grade petroleum ether and acetone were purchased from Thermo Scientific (Waltham, MA, USA) and butylated hydroxytoluene (BHT) from MP Biomedicals (Santa Ana, CA, USA). Authentic reference standards were purchased from Fisher Scientific, Sigma-Aldrich, Alfa Aesar, Toronto Research Chemicals (Toronto, Ontario, Canada), Apollo Chemical (Burlington, NC, USA), and TCI Chemicals (Portland, OR, USA). These include β-carotene; lutein; lycopene; 4-hydroxybenzoic acid (4-HBA); 3,4-dihydroxybenzoic acid; 4-hydroxycinnamic acid; 3,4-dihydroxycinnamic acid; 4-hydroxy-3-methoxycinnamic acid; 3,4,5-trihydroxybenzoic acid; 4-hydroxyhippuric acid; dihydroresveratrol; kaempferol; catechin; epicatechin; gallocatechin; epigallocatechin; resveratrol 3-*O*-sulfate; 4-*O*-caffeoylquinic acid; 3-*O*-caffeoylquinic acid; 5-*O*-caffeoylquinic acid; epigallocatechin gallate; urolithin A; taxifolin; 3,4-dihydroxybenzaldehyde; 3-(4-hydroxy-3-methoxyphenyl)propionic acid; myricetin; isourolithin A; 3-(3,4-dihydroxyphenyl)propionic acid; dihydrocaffeic acid 3-*O*-sulfate; naringenin; ferulic acid 4-*O*-sulfate; benzoic acid 4-*O*-glucuronide; caffeic acid 4-*O*-glucuronide; caffeic acid 3-*O*-glucuronide; ferulic acid 4-*O*-glucuronide; dihydroferulic acid 4-*O*-glucuronide; isourolithin A 9-glucuronide; 3,4-dihydroxyphenylacetic acid; hesperetin 3-*O*-glucuronide; and ethyl gallate.

### 2.2 Study design and participants

The present study was a secondary analysis of breastfeeding women that were enrolled in a within-subject intervention pilot study (NCT03744429). This study was designed with the intent of leveraging results to design a more comprehensive intervention for evaluation of relationships between maternal dietary and non-dietary factors, HM bioactive composition, and infant developmental outcomes. Healthy women with obesity (BMI = 30–50 kg/m^2^) at 4 to 5 months postpartum who were exclusively breastfeeding were recruited from the Little Rock, Arkansas community between April 2019 and February 2020, as described previously (26). All procedures within the study were approved by the University of Arkansas for Medical Sciences Institutional Review Board (Protocol #228407). Participants underwent a written, informed consent process at enrollment. Enrollment criteria have been described previously (26). Ninety women were screened, 28 were eligible, and 16 (BMI = 30–50 kg/m^2^) were enrolled. Due to three participants not completing all study visits, 13 participants completed all study visits and were included for analysis. Maternal race and ethnicity, age, and infant sex were self-reported at screening. The study consisted of three visits: enrollment, 2 weeks, and 4 weeks following the introduction of the dietary intervention. The enrollment visit is considered pre-intervention and the 4-week time point is considered post-intervention.

### 2.3 3-day food records

Prior to initiation of the dietary intervention, participants recorded all food, beverages, supplements, and medications consumed over 3 days (two weekdays and one weekend day). For the entirety of the intervention, participants recorded all food, beverages, dietary supplements, medications, and any substitutions made to the meal plan. Dietary intake information was analyzed by trained staff with the Nutrition Data System for Research (NDSR, Nutrition Coordinating Center, University of Minnesota, MN, USA) software. Average daily intake at pre-intervention and during the intervention (average of wk. 1–wk. 4) for carotenoids, polyphenols and phytochemical-rich food groups, were estimated from NDSR diet records. Intake of food groups and the carotenoids β-cryptoxanthin, lutein+zeaxanthin, lycopene, α-carotene, and β-carotene were provided from NDSR. Polyphenol intake was estimated using a database developed from Phenol-Explorer (37) and polyphenol concentrations reported in published literature.

### 2.4 Dietary intervention

The intervention consisted of a Mediterranean-style diet pattern with macronutrient distribution (20–35% of calories from fat, 45–65% carbohydrates, 10–35% protein) and caloric intake that met recommendations from the Dietary Guidelines for Americans (DGA). Participants were provided with all lunches and dinners (fresh-packaged meals) weekly throughout the 4-week intervention by Trifecta Nutrition (Sacramento, CA, USA). Breakfast and snacks consisting of breakfast sandwiches, oatmeal, walnuts, granola bars, Greek yogurt, and fruits were provided by the research team. Additionally, participants were provided with Palermo extra virgin olive oil to add to meals and were instructed to buy 1% low fat milk to drink or add to fruit smoothies. Adherence to the dietary intervention was greater than 80%, as reported previously (26).

### 2.5 Human milk collection

Prior to each visit, mothers collected HM over a 24-h time period. Mothers were asked to collect milk from each feed with the option to feed their infant expressed milk or feed from one breast and pump from the other. If the latter option was selected, the pumped breast would be alternated at every feed time. Following collection, expressed milk was gently inverted and 4 ml was aliquoted into provided polypropylene tubes. Human milk was stored at 4°C until the full 24-h was completed. Pooled milk samples were aliquoted and stored at −80°C until further analysis.

### 2.6 Extraction and analysis of carotenoids from human milk by HPLC

Concentrations of carotenoids in human milk were determined by HPLC, as described previously (6, 38). Carotenoids were separated on a YMC C30 (2.0 × 150 mm) column (YMC Kyoto, Japan) with diode array detection. Quantification of carotenoids was accomplished with authentic carotenoid standards for β-carotene, lutein, and lycopene via calibration curves extracted at 450 nm. Identification of β-cryptoxanthin, α-carotene, and zeaxanthin was based on comparison of absorption spectra and elution profiles of previous results using similar C30 chromatography (5). Concentrations of β-cryptoxanthin and α-carotene were estimated using the calibration curve for β-carotene. Zeaxanthin content was estimated based on the calibration curve for lutein.

### 2.7 Extraction and analysis of polyphenol metabolites from human milk by UPLC-MS/MS

Extraction methodologies for polyphenol metabolites were adapted from Henning et al. (17). Aliquots of HM (250 μl) were extracted with 1 ml of acetonitrile:formic acid (98:2) three times. Dried extracts were reconstituted in 400 μl of water:formic acid (99.9:0.1), filtered on AcroPrep™ 96-well filter plates (1 ml, 0.45 μm Supor membrane, Cytiva, Marlborough, MA, USA), and analyzed by UPLC-MS/MS. Final concentrations were adjusted by recovery of the internal standard taxifolin, which was added to all milk samples as internal standard at the beginning of analysis. Phenolic compounds and metabolites were resolved with an Acquity UPLC BEH C18 column using a Waters Acquity Premier UPLC system equipped with a XEVO TQS-micro mass spectrometer (Waters, Milford, MA, USA) as previously described (39). Multiple reaction monitoring (MRM) was used to identify and quantify 35 individual phenolic and metabolite compounds (Supplementary Table S1). Validation parameters are reported in Supplementary Table S2.

### 2.8 Human milk oligosaccharide composition and maternal FUT2 phenotype

Concentrations of the 19 most abundant HMOs (3) were determined by HPLC, as previously described (27). Individual HMOs were separated on an amide-80 column (2 μm particle size, 2 mm ID, 15 cm length) with fluorescent detection. Final concentrations were adjusted by recovery of the internal standard raffinose, which was added to all milk samples as internal standard at the beginning of analysis. Maternal FUT2 secretor phenotype was determined by presence (secretor) or near-absence (non-secretor) of 2′-fucosyllactose (2’FL) in HM (<100 nmol/ml).

### 2.9 Statistical analysis

Using macronutrient (fat, protein, carbohydrates) content of 24-h pooled samples measured with a Miris HM Analyzer (Miris, Uppsala, Sweden), HM carotenoid concentrations were expressed on a lipid basis (nmol/g fat). Polyphenol metabolites (nmol/l) and HMOs (nmol/ml) were expressed on a volume basis. For statistical analyses, concentrations that were not detected (ND) and below the limit of detection (<LOD) were imputed as 1/10 and 1/5 of the LOD, respectively. Data in tables represents raw data, while data in figures represents data used for statistical analyses (i.e., with imputed values). Repeated measures ANOVA was used to determine the main effects of time of the dietary intervention (Pre vs. Wk4) and the time-by-secretor status (secretor vs. non-secretor) interaction for diet and HM bioactive composition. Due to skewness and non-normality of HM bioactives (carotenoids, HMOs, polyphenol metabolites), ANOVA models and post-hoc analyses were evaluated after rank-based transformation was carried out using the *nparLD* R package (version 2.2) (40). ANOVA models and post-hoc analyses (Tukey HSD) of diet composition were evaluated using the *lmerTest (version 3.1.3)* (41) and *emmeans (version 1.10.0)* (42) R packages. An exploratory analysis of associations between HM bioactives for secretors and non-secretors was carried out using repeated measures correlations using the *rmcorr* (version 0.6.0) (43) R package. *p*-values for the correlation analysis were FDR-adjusted. All statistical analyses were carried out using R statistical software (version 4.3.2) (44). Sensitivity analyses were carried out for measurements identified as extreme outliers (3 times above the upper quartile or 3 times below the lower quartile).

## 3 Results

### 3.1 Maternal baseline characteristics

Participants were on average 33 ± 4 years of age (secretors: 35 ± 4; non-secretors: 31 ± 3) and 77% of participants were of non-Hispanic, Caucasian descent (secretors: 67%; non-secretors: 86%; Table 1**)**. All participants were with obesity at baseline (all participants mean body mass index: 36 ± 5 kg/m^2^; secretors: 35 ± 5 kg/m^2^; non-secretors: 37 ± 5 kg/m^2^).

**Table 1:** Baseline characteristics of lactating women with obesity upon enrollment.^a^

Characteristic	All participants (*N* = 13)	Secretors (*N* = 6)	Non-secretors (*N* **=** 7)	*p*
Maternal age, y^*^	33 ± 4	35 ± 4	31 ± 3	0.12
Maternal height, cm^*^	164 ± 5	164 ± 5	164 ± 6	0.95
Maternal weight, kg^*^	97 ± 15	93 ± 15	99 ± 16	0.49
Maternal BMI^*^	36 ± 5	35 ± 5	37 ± 5	0.42
Maternal race^‡^				0.71
African American Non-Hispanic	2 (15%)	1 (17%)	1 (14%)	
White Hispanic	1 (8%)	1 (17%)	0 (0%)	
White Non-Hispanic	10 (77%)	4 (67%)	6 (86%)	
Maternal education^‡^				1.00
High school/specialized training	3 (23%)	1 (17%)	2 (29%)	
Partial college/college degree	7 (54%)	3 (50%)	4 (57%)	
Graduate training/degree	3 (23%)	2 (33%)	1 (14%)	
Household income^‡^				1.00
<$40,000	5 (38%)	2 (33%)	3 (43%)	
$40,000–$70,000	5 (38%)	3 (50%)	2 (29%)	
>$70,000	3 (23%)	1 (17%)	2 (29%)	
Infant sex^‡^				0.69
Female	5 (38%)	2 (33%)	3 (43%)	
Male	8 (62%)	4 (67%)	4 (57%)	

*p-values represent comparisons between secretors and non-secretors. BMI: body mass index.*

a Data are presented as mean ± SD or number (%). Statistical analyses were performed using:

* Student *t*-test, or.

‡ Fisher’s exact test.

### 3.2 Maternal dietary intake before and during the intervention

During the intervention (wk 1 to wk. 4), intake of whole grain (+75%, *p* < 0.001), fruit (+225%, *p* < 0.001), and vegetable (+136%, *p* < 0.001) intake significantly increased for all participants, regardless of secretor status (Table 2). With the exception of lycopene, intake of all other phytochemicals increased, including *β*-carotene (+841%, *p* < 0.001), β-cryptoxanthin (+232%, *p* < 0.001), and lutein+zeaxanthin (+290%, *p* < 0.001), and total polyphenols (+114%, *p* < 0.001). The observed decrease in lycopene intake (−58%, *p* < 0.001) was due to three participants that consumed greater amounts of lycopene derived from intake of tomato products at baseline. After exclusion of these participants, there was no significant change in lycopene intake (Pre-intervention: 3,097 ± 2,194 μg/d; Intervention: 2,431 ± 1,840 μg/d; *p* = 0.34).

**Table 2:** Daily intake of polyphenols, carotenoids, and phytochemical-rich food groups before and during Mediterranean dietary intervention.^a^

		Pre-intervention^b^			Intervention^c^		
	NHANES average daily intake^d^	All participants	Secretors	Non-secretors	All participants	Secretors	Non-secretors
Phytochemical intake							
Total polyphenols (mg)	1,250	1,003 ± 438	918 ± 400	1,076 ± 487	2,145 ± 818^*^	2,208 ± 880^*^	2,094 ± 775^*^
β-cryptoxanthin (μg)	70	74 ± 114	37 ± 26	106 ± 151	246 ± 236^*^	263 ± 295^*^	231 ± 176
Lutein + Zeaxanthin (μg)	1,400	1,409 ± 1,361	2,121 ± 1,770	798 ± 387	5,494 ± 1,742^*^	5,157 ± 1,381^*^	5,771 ± 1,972^*^
Lycopene (μg)	5,500	5,830 ± 5,613	5,151 ± 4,573	6,413 ± 6,687	2,431 ± 1,840^*^	2,986 ± 1,772^*^	1,975 ± 1,798^*^
α-carotene (μg)	350	245 ± 209	238 ± 174	251 ± 249	934 ± 526^*^	887 ± 424^*^	973 ± 601^*^
β-carotene (μg)	1,800	1,857 ± 1,259	2,414 ± 1,565	1,380 ± 744	17,471 ± 8,090^*^	19,886 ± 7,430^*^	15,487 ± 8,195^*^
Total carotenoids (μg)	9,100	9,171 ± 5,582	9,724 ± 5,557	8,697 ± 6,001	25,642 ± 8,277^*^	28,292 ± 7,618^*^	23,465 ± 8,288^*^
Food group servings							
Whole grains (1 serving = 1/2 cup)	0.8	2.0 ± 1.3	1.5 ± 1.1	2.3 ± 1.4	3.5 ± 0.9^*^	3.9 ± 0.9^*^	3.2 ± 0.9^*^
Fruits (1 serving = 1/2 cup)	0.9	1.2 ± 1.8	0.9 ± 1.2	1.5 ± 2.2	3.9 ± 2.3^*^	3.8 ± 2.0^*^	4.0 ± 2.6^*^
Vegetables (1 serving = 1/2 cup)	1.5	2.8 ± 0.9	2.9 ± 1.0	2.7 ± 0.8	6.6 ± 1.4^*^	6.6 ± 1.3^*^	6.6 ± 1.5^*^

a Data represented as mean ± SD.

b Average daily intake calculated from 3-day diet records.

c Average daily intake calculated from daily diet records collected during the 4-week dietary intervention.

d Average daily intake for U.S. adults based on the National Health and Nutrition Examination Survey (NHANES) 2007–2016 (77), 2009–2018 (45), 2017–2018 (78), and 2013–2016 (79) for polyphenol, carotenoid, whole grain, and fruit and vegetable intake, respectively.

* Significantly different from pre-Intervention (*p* < 0.05).

### 3.3 Association of maternal diet and secretor status with human milk bioactive composition

#### 3.3.1 Carotenoids

Concentrations of major carotenoid species in HM before and after the dietary intervention are summarized in Table 3. Because significant time-by-secretor status interactions were not observed, only main effects of time were analyzed. Despite significant increases in dietary carotenoid intake, significant changes in concentrations of carotenoids in HM were not observed. Analysis of dietary intake revealed wide variation in carotenoid intake during the intervention (Supplementary Table S3), despite consuming amounts greater than NHANES average daily intakes for U.S. adults (45) on at least 60% of intake days during the intervention. The largest fluctuations in daily intakes were observed for β-carotene (IQR: 2,767–30,342 μg/d) as a result of daily participant food choices.

**Table 3:** Concentrations of carotenoids (nmol/g fat) in human milk samples of lactating women before and after 1 month Mediterranean-style dietary intervention.

	Pre-intervention						Post-intervention					
	All participants		Secretors		Non-secretors		All participants		Secretors		Non-secretors	
Carotenoid	*n* ^a^	Mean ± SD (range)	*n*	Mean ± SD (range)	*n*	Mean ± SD (range)	*n*	Mean ± SD (range)	*n*	Mean ± SD (range)	*n*	Mean ± SD (range)
Lutein	12	3.7 ± 2.7 (0.5–7.7)	6	3.6 ± 2.5 (1.2–6.9)	6	3.8 ± 3.2 (0.5–7.7)	8	4.3 ± 3.8 (0.4–8.8)	5	4.3 ± 4.2 (0.4–8.8)	3	4.3 ± 3.9 (0.5–8.4)
Zeaxanthin	11	2.0 ± 2.0 (0.2–6.8)	6	1.9 ± 1.4 (0.4–4.1)	5	2.0 ± 2.8 (0.2–6.8)	7	2.6 ± 2.4 (0.4–6.9)	4	2.9 ± 2.9 (0.4–6.9)	3	2.1 ± 1.9 (0.6–4.2)
β-Cryptoxanthin	11	2.3 ± 1.9 (0.2–6.0)	5	2.8 ± 0.7 (0.8–4.9)	6	1.9 ± 2.1 (0.2–6.0)	12	2.4 ± 1.8 (0.3–6.7)	6	2.7 ± 2.5 (0.3–6.7)	6	2.2 ± 0.7 (1.1–3.0)
α-Carotene	5	4.8 ± 8.1 (0.7–19.3)	3	1.4 ± 0.3 (1.2–1.7)	2	10.0 ± 13.2 (0.7–19.3)	8	4.1 ± 3.9 (0.7–10.5)	5	3.7 ± 3.8 (0.7–10.2)	3	4.9 ± 4.9 (1.7–10.5)
β-Carotene	13	40.8 ± 95.3 (0.2–314.7)	6	33.6 ± 70.4 (0.4–177.0)	7	47.0 ± 118.0 (0.2–314.7)	13	93.7 ± 192.3 (0.3–541.2)	6	106.4 ± 214.3 (0.3–541.2)	7	82.8 ± 188.0 (1.0–507.8)
Lycopene	12	3.8 ± 2.5 (0.6–8.4)	6	4.0 ± 3.2 (0.6–8.4)	6	3.5 ± 1.7 (2.1–6.0)	12	4.7 ± 2.5 (2.0–10.0)	5	5.0 ± 3.1 (2.0–10.0)	7	4.5 ± 2.2 (0.6–8.4)
Total carotenoids	12	53.1 ± 106.4 (1.6–360.3)	6	46.1 ± 76.6 (5.8–201.2)	7	59.2 ± 132.9 (1.6–360.3)	13	106.8 ± 191.0 (2.6–551.3)	6	121.9 ± 212.0 (2.6–551.3)	7	93.9 ± 187.4 (4.8–516.4)

a *n* = number of samples in which the compound was detected and quantified. *N* = 13 for all participants (6 secretors, 7 non-secretors).

**Figure 1:**
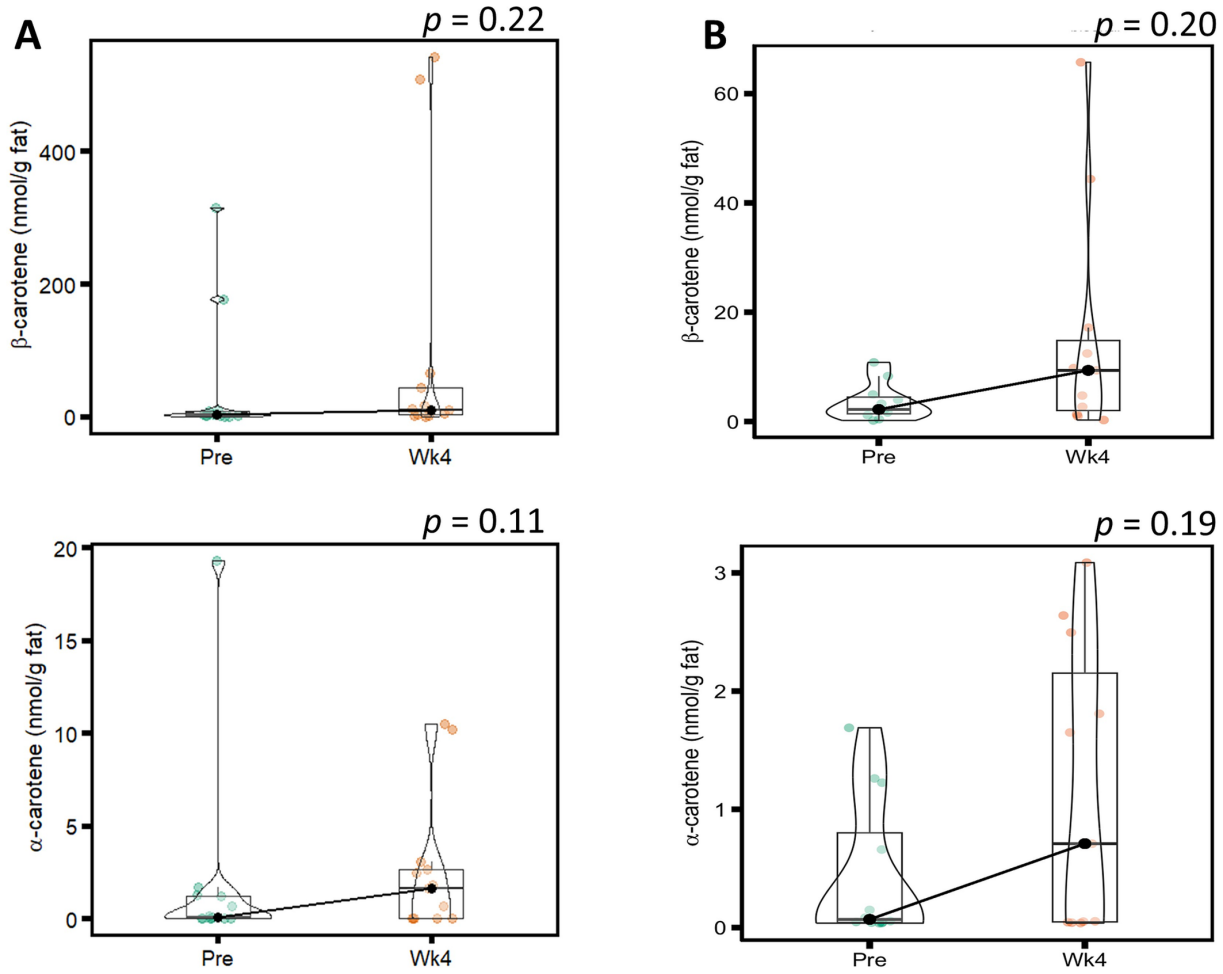
Violin plot with boxplot overlay of sensitivity analysis for ɑ-carotene and β-carotene (nmol/g fat) before (Pre) and after (Wk4) the intervention for **(A)** all participants and **(B)** after exclusion of extreme outliers. Statistics determined using 2-way repeated measures ANOVA models of time by secretor status following rank-based transformation of data. *p*-values shown represent the main effects of time in the ANOVA models since the interaction term was not significant.

Concentrations of α-carotene and β-carotene concentrations in HM have rarely been reported to exceed ~10 nmol/g fat, even with large supplementation doses of α-carotene and β-carotene (6, 19–21). Because extreme outliers were observed in α-carotene and β-carotene concentrations, a sensitivity analysis was carried out (Figure 1). No significant changes in the concentrations of α-carotene and β-carotene were observed when all data was included nor after exclusion of extreme outliers. A summary of ANOVA model *p*-values for analyses of carotenoids and sensitivity analysis for α-carotene and β-carotene are shown in Supplementary Table S4. The complete dataset for individual carotenoid concentrations in all HM samples is in Table A in Supplementary File S1.

#### 3.3.2 Polyphenols

Concentrations of polyphenol metabolites in HM before and after the dietary intervention are summarized in Table 4. Unlike carotenoid intake, less variability in day-to-day polyphenol intake was observed across all intervention intake days (IQR: 1,249–2,642 mg/d, Supplementary Table S3). Time-by-secretor status interactions were observed for 4-HBA (*p* = 0.08) and ferulic acid (*p* = 0.02). Post-hoc analyses revealed that 4-HBA and ferulic acid significantly increased over the intervention time frame for secretors (4-HBA: +227%, *p* = 0.02; ferulic acid: +488%, *p* = 0.006), but not for non-secretors (4-HBA: +17%; ferulic acid: −7%). P-coumaric acid significantly increased over time (median at Pre-Intervention: 0 nmoL/L; median at Post-Intervention: 2.58 nmoL/L; *p* = 0.02) with no time-by-secretor interaction. A summary of ANOVA model *p*-values for analyses of polyphenol metabolites are shown in Supplementary Table S4. The complete dataset for individual polyphenol metabolite concentrations in all HM samples is in Table B in Supplementary File S1.

**Table 4:** Concentrations of polyphenol metabolites (nmol/l) in human milk samples of lactating women before and after 1 month Mediterranean-style dietary intervention.^a^

	Pre-intervention						Post-intervention					
	All participants		Secretors		Non-secretors		All participants		Secretors		Non-secretors	
Polyphenol	*n* ^b^	Mean ± SD (range)	*n*	Mean ± SD (range)	*n*	Mean ± SD (range)	n	Mean ± SD (range)	*n*	Mean ± SD (range)	*n*	Mean ± SD (range)
Flavonoids												
Naringenin	3	5.4 ± 2.5 (3.8–8.2)	1	8.2	2	3.9 ± 0.1 (3.8–4.0)	2	5.5 ± 0.8 (4.9–6.0)	0	ND	2	5.5 ± 0.8 (4.9–6.0)
Kaempferol	0	ND	0	ND	0	ND	0	ND	0	ND	0	ND
Myricetin	12	26.1 ± 3.8 (20.6–32.3)	6	25.8 ± 3.7 (22.8–32.3)	6	26.4 ± 4.3 (20.6–31.6)	11	26.7 ± 6.2 (21.0–38.7)	6	27.6 ± 7.7 (21.0–38.7)	5	25.6 ± 4.3 (21.7–32.3)
Hesperetin 3-*O*-glucuronide	5	51.3 ± 44.7 (27.7–130.8)	3	32.4 ± 5.8 (27.7–38.9)	2	79.5 ± 72.6 (28.2–130.8)	8	54.6 ± 75.9 (22.2–241.7)	3	32.6 ± 9.9 (26.5–44.1)	5	67.7 ± 97.3 (22.2–241.7)
Catechin	0	ND	0	ND	0	ND	0	ND	0	ND	0	ND
Epicatechin	0	ND	0	ND	0	ND	0	ND	0	ND	0	ND
Gallocatechin	1	6.6	1	6.6	0	ND	2	4.7 ± 1.2 (3.8–5.5)	2	4.7 ± 1.2 (3.8–5.5)	0	ND
Epigallocatechin	0	ND	0	ND	0	ND	0	ND	0	ND	0	ND
Epigallocatechin gallate	0	ND	0	ND	0	ND	0	ND	0	ND	0	ND
Total flavonoids	13	45.5 ± 40.6 (3.8–165.9)	6	44.5 ± 19.5 (23.2–68.3)	7	46.4 ± 54.6 (3.8–165.9)	13	57.7 ± 64.1 (21.0–263.6)	6	45.4 ± 19.7 (21.0–71.5)	7	68.3 ± 87.3 (21.7–263.6)
Hydroxybenzoic acids												
3,4-Dihydroxybenzaldehyde	0	ND	0	ND	0	ND	0	ND	0	ND	0	ND
3,4-Dihydroxybenzoic acid	1	15.0	1	15.0	0	ND	3	22.3 ± 8.3 (15.1–31.4)	1	20.3	2	23.2 ± 11.5 (15.1–31.4)
4-Hydroxybenzoic acid^†^	13	93.8 ± 72.9 (26.2–240.2)	6	97.5 ± 88.6 (37.6–240.2)	7	90.6 ± 63.7 (26.2–217.6)	13	125.8 ± 91.4^*^ (24.5–273.3)	6	158.1 ± 98.3^*^ (52.2–270.6)	7	98.1 ± 82.0 (24.5–273.3)
Gallic acid	8	446.9 ± 230.1 (223.4–805.4)	4	523.4 ± 309.6 (223.4–805.4)	4	370.5 ± 110.1 (300.8–532.7)	6	320.7 ± 87.4 (245.9–436.2)	3	322.1 ± 98.8 (264.9–436.2)	3	319.3 ± 96.6 (245.9–428.7)
Benzoic acid 4-*O*-glucuronide	1	12.4	0	ND	1	12.4	0	ND	0	ND	0	ND
Total HBAs	13	370.9 ± 299.1 (26.2–850.2)	6	448.9 ± 361.1 (37.6–850.2)	7	304.1 ± 242.9 (26.2–642.9)	13	278.9 ± 173.5 (63.6–535.8)	6	322.5 ± 202.8 (79.0–535.8)	7	241.5 ± 149.6 (63.6–474.2)
Hydroxycinnamic acids												
3-*O*-Caffeoylquinic acid	0	ND	0	ND	0	ND	0	ND	0	ND	0	ND
4-*O*-Caffeoylquinic acid	0	ND	0	ND	0	ND	0	ND	0	ND	0	ND
5-*O*-Caffeoylquinic acid	0	ND	0	ND	0	ND	0	ND	0	ND	0	ND
*p*-Coumaric acid	4	3.0 ± 0.9 (1.8–3.7)	3	2.9 ± 1.0 (1.8–3.7)	1	3.4	9	4.9 ± 4.4^*^ (1.4–15.2)	4	6.3 ± 5.9 (2.9–15.2)	5	3.8 ± 2.8^*^ (1.4–8.3)
Caffeic acid	0	ND	0	ND	0	ND	0	ND	0	ND	0	ND
Ferulic acid^†^	9	6.4 ± 3.5 (2.2–13.7)	3	6.6 ± 6.2 (2.2–13.7)	6	6.3 ± 2.1 (4.2–9.7)	12	7.2 ± 4.4^*^ (2.5–17.8)	5	9.0 ± 5.2^*^ (4.6–17.8)	7	5.8 ± 3.7 (2.5–13.0)
Ferulic acid 4-*O*-sulfate	7	4.3 ± 1.4(2.5–6.7)	3	3.3 ± 0.7 (2.5–3.9)	4	5.0 ± 1.4 (3.7–6.7)	4	3.8 ± 1.1 (3.0–5.5)	2	3.4 ± 0.2 (3.2–3.5)	2	4.2 ± 1.7 (3.0–5.5)
Ferulic acid 4-*O*-glucuronide	0	ND	0	ND	0	ND	0	ND	0	ND	0	ND
Caffeic acid 3-*O*-glucuronide	0	ND	0	ND	0	ND	0	ND	0	ND	0	ND
Caffeic acid 4-*O*-glucuronide	13	8.0 ± 2.3 (5.0–12.6)	6	7.3 ± 1.6 (5.3–9.7)	7	8.6 ± 2.8 (5.0–12.6)	13	9.4 ± 3.5 (4.3–14.4)	6	9.7 ± 4.1 (4.3–14.4)	7	9.1 ± 3.3 (4.5–12.5)
Total HCAs	13	15.7 ± 6.5 (8.8–29.0)	6	13.7 ± 5.8 (8.8–22.7)	7	17.3 ± 7.1 (10.2–29.0)	13	20.6 ± 10.0 (10.4–47.4)	6	22.6 ± 13.0 (11.7–47.4)	7	18.9 ± 7.0 (10.4–28.9)
Hydroxyphenylpropionic acids												
Dihydrocaffeic acid	8	15.4 ± 7.1 (7.2–25.7)	5	16.2 ± 8.3 (7.2–25.7)	3	14.0 ± 5.9 (9.3–20.7)	4	13.1 ± 7.0 (8.5–23.5)	1	11.0	3	13.8 ± 8.4 (8.5–23.5)
Dihydroferulic acid	11	50.9 ± 12.7 (34.7–85.0)	5	54.2 ± 17.4 (44.3–85.0)	6	48.2 ± 7.8 (34.7–56.8)	9	46.6 ± 5.5 (39.7–55.0)	5	46.6 ± 5.9 (40.9–55.0)	4	46.5 ± 5.9^*^ (39.7–53.5)
Dihydrocaffeic acid 3-*O*-sulfate	1	2.8	1	2.8	0	ND	0	ND	0	ND	0	ND
Dihydroferulic acid 4-*O*-glucuronide	0	ND	0	ND	0	ND	0	ND	0	ND	0	ND
Total OH-PPAs	13	52.8 ± 24.5 (7.2–96.0)	6	59.2 ± 30.4 (7.2–96.0)	7	47.3 ± 18.7 (9.3–69.8)	9	52.4 ± 10.2 (39.7–71.9)	5	48.8 ± 5.3 (40.9–55.0)	4	56.9 ± 13.9 (39.7–71.9)
Other phenolic acids												
3,4-Dihydroxyphenylacetic acid	0	ND	0	ND	0	ND	1	322.3	1	322.3	0	ND
4-Hydroxyhippuric acid	13	146.4 ± 91.4 (55.0–328.5)	6	145.7 ± 105.1 (61.1–328.5)	7	147.0 ± 86.6 (55.0–319.0)	13	165.2 ± 118.1 (36.2–466.5)	6	194.8 ± 148.6 (55.2–466.5)	7	139.8 ± 88.8 (36.2–310.1)
Total phenolic acids	13	585.8 ± 329.8 (140.8–1,001.6)	6	667.5 ± 395.0 (162.3–1,001.6)	7	515.8 ± 274.0 (140.8–865.3)	13	525.8 ± 226.3 (256.5–1,071.0)	6	634.4 ± 263.0 (264.1–1,071.0)	7	432.7 ± 151.1 (256.5–667.1)
Stilbenes												
Resveratrol 3-*O*-sulfate	5	3.2 ± 0.3 (2.8–3.6)	2	3.0 ± 0.2 (2.9–3.2)	3	3.3 ± 0.4 (2.8–3.6)	5	3.3 ± 0.4 (2.8–3.8)	4	1.4 ± 0.4 (2.8–3.8)	1	3.0
Dihydroresveratrol	0	ND	0	ND	0	ND	0	ND	0	ND	0	ND
Total stilbenes	13	3.2 ± 0.3 (2.8–3.6)	2	3.0 ± 0.2 (2.9–3.2)	3	3.3 ± 0.4 (2.8–3.6)	5	3.3 ± 0.4 (2.8–3.8)	4	3.4 ± 0.4 (2.8–7.8)	1	3.0
Urolithins												
Isourolithin A	0	ND	0	ND	0	ND	2	3.2 ± 0.4 (2.9–3.4)	2	3.2 ± 0.4 (2.9–3.4)	0	ND
Urolithin A	1	1.4	0	ND	1	1.4	0	ND	0	ND	0	ND
Isourolithin A glucuronide	0	ND	0	ND	0	ND	1	17.5	1	17.5	0	ND
Total urolithins	1	1.4	0	ND	1	1.4	2	7.8 ± 12.7 (2.9–20.9)	2	7.8 ± 12.7 (2.9–20.9)	0	ND
Total polyphenols	13	633 ± 349 (145–1,065)	6	713 ± 397 (188–1,065)	7	564 ± 317 (145–1,034)	13	587 ± 255 (292–1,134)	6	686 ± 274 (292–1,134)	7	501 ± 221 (292–931)

a Statistics determined using 2-way repeated measures ANOVA models of time by secretor status following rank-based transformation.

† Polyphenol metabolites that demonstrated either a statistical trend (*p* < 0.1) or significance (*p* < 0.05) for the interaction term in ANOVA model.

b *n* = number of samples in which the compound was detected and quantified. *N* = 13 for all participants (6 secretors, 7 non-secretors).

* Significant difference (*p* < 0.05) between Pre-Intervention and Post-Intervention.

#### 3.3.3 Human milk oligosaccharides

Concentrations of HMOs before and after the dietary intervention are summarized in Table 5 and were reported previously without stratification by secretor status (26). A significant time-by-secretor status interaction was observed for lacto-N-tetraose (LNT), lacto-N-fucopentaose II (LNFP II), difucosyllacto-N-tetraose (DFLNT), and disialyllacto-N-tetraose (DSLNT). Post-hoc analyses demonstrated a significant increase in concentrations of DSLNT over the intervention timeframe for secretors (+38%, *p* = 0.0008) and decreases in concentrations of LNT (−15%, *p* < 0.001), LNFP II (−20%, *p* = 0.008), and DFLNT (−96%, *p* < 0.001) for non-secretors. A summary of ANOVA model *p*-values for analysis of HMOs are shown in Supplementary Table S4.

**Table 5:** Concentrations of oligosaccharides (HMOs, nmol/ml) in human milk samples of lactating women before and after 1 month Mediterranean-style dietary intervention.^a^

	Pre-intervention			Post-intervention		
	All participants	Secretors	Non-secretors	All participants	Secretors	Non-secretors
HMO	Mean ± SD	Mean ± SD	Mean ± SD	Mean ± SD	Mean ± SD	Mean ± SD
Lacto-N-tetraose (LNT) ^†^	758.8 ± 528.2	656.0 ± 530.0	848.7 ± 551.0	738.6 ± 534.9	789.2 ± 590.0	695.3 ± 526.8^*^
Lacto-N-neotetraose (LNnT)	56.4 ± 32.4	61.1 ± 42.9	51.7 ± 20.2	51.9 ± 35.9	53.0 ± 24.7	50.9 ± 45.5
Lacto-N-hexaose (LNH)	27.5 ± 20.2	24.2 ± 12.4	29.8 ± 25.1	22.3 ± 10.0	23.4 ± 13.8	21.6 ± 6.2
Neutral HMOs	836.4 ± 553.5	735.3 ± 574.6	922.8 ± 564.6	812.8 ± 536.0	865.6 ± 617.1	767.5 ± 502.2
2′-Fucosyllactose (2’FL)	1,753.4 ± 2,038.9	3,746.5 ± 1,060.0	45.0 ± 27.3	1,906.3 ± 2,601.1	4,079.9 ± 2,389.1	43.1 ± 19.6
3-Fucosyllactose (3FL)	2,671.3 ± 1,715.8	1,239.8 ± 975.3	3,898.3 ± 1,135.6	2,626.0 ± 1,476.5	1,388.2 ± 1,104.8	3,687.0 ± 705.2
difucosyllactose (DFLac)	35.2 ± 38.5	68.4 ± 33.0	6.7 ± 3.3	31.8 ± 41.5	69.8 ± 40.6	4.7 ± 2.4
Lacto-N-fucopentaose I (LNFP I)	345.8 ± 331.6	602.6 ± 336.0	125.7 ± 57.9	366.7 ± 369.2	665.8 ± 350.0	110.4 ± 66.1
Lacto-N-fucopentaose II (LNFP II) ^†^	882.3 ± 513.1	517.0 ± 273.6	1,195.4 ± 465.1	787.6 ± 468.7	518.8 ± 239.6	1,018.0 ± 507.3^*^
lacto-N-fucopentaose III (LNFP III)	11.9 ± 8.3	12.3 ± 8.1	11.5 ± 9.0	7.3 ± 5.6^*^	10.1 ± 6.9	4.9 ± 2.9^*^
Difucosyllacto-N-tetraose (DFLNT)^†^	120.0 ± 97.4	116.0 ± 138.6	123.5 ± 54.0	48.7 ± 62.2^*^	71.6 ± 76.9	29.0 ± 42.8^*^
Fucosyllacto-N-hexaose (FLNH)	80.4 ± 38.7	70.5 ± 29.0	87.5 ± 45.2	83.0 ± 58.1	77.1 ± 39.3	88.0 ± 73.5
Difucosyllacto-N-hexaose (DFLNH)	21.3 ± 18.7	30.3 ± 18.6	13.6 ± 16.2	19.8 ± 15.3	31.7 ± 13.8	9.7 ± 6.9
Neutral fucosylated HMOs	6,184.4 ± 1,145.2	6,717.9 ± 522.8	5,727.0 ± 1,366.1	5,644.3 ± 1,180.9	6,390.3 ± 996.1	5,111.5 ± 1,049.0
3′-sialyllactose (3’SL)	234.6 ± 162.3	367.9 ± 149.9	120.4 ± 31.4	227.2 ± 154.9	359.5 ± 130.3	113.8 ± 36.6
6′-sialyllactose (6’SL)	148.5 ± 44.6	159.5 ± 36.0	139.1 ± 51.8	131.8 ± 87.2	132.4 ± 56.9	131.3 ± 111.9
Sialyllacto-N-tetraose b (LSTb)	59.0 ± 40.6	46.1 ± 31.8	70.0 ± 46.3	54.5 ± 35.6	50.5 ± 27.8	58.0 ± 43.1
Sialyllacto-N-tetraose c (LSTc)	21.2 ± 12.8	24.0 ± 17.9	18.8 ± 6.6	23.2 ± 19.7	27.7 ± 22.3	14.3 ± 11.0
Disialyllacto-N-tetraose (DSLNT)^†^	65.5 ± 38.7	70.8 ± 54.8	61.0 ± 20.8	76.1 ± 47.8	99.1 ± 59.0^*^	56.3 ± 26.1
Disialyllacto-N-hexaose (DSLNH)	39.6 ± 15.6	53.1 ± 9.4	28.0 ± 8.7	33.8 ± 28.9	43.0 ± 19.5	25.9 ± 34.6
Fucodisialyllacto-N-hexaose (FDSLNH)	66.5 ± 44.5	55.5 ± 37.3	76.1 ± 50.7	66.3 ± 38.5	58.3 ± 29.7	73.1 ± 46.0
Acidic (sialylated) HMOs	806.6 ± 278.4	956.2 ± 282.0	678.3 ± 217.2	861.8 ± 332.6	970.9 ± 222.8	768.3 ± 397.2
Total HMOs	7,441.4 ± 1,260.4	7,989.5 ± 971.5	6,971.6 ± 1,353.9	6,974.8 ± 1,583.0	7,813.9 ± 1,517.8	6,375.4 ± 1,432.7

a *N* = 13 (6 secretors and 7 non-secretors). Statistics determined using 2-way repeated measures ANOVA models of time by secretor status following rank-based transformation.

† HMOs that demonstrated either a statistical trend (*p* < 0.1) or significance (*p* < 0.05) for the interaction term in ANOVA model.

* Significant difference (*p* < 0.05) between Pre-Intervention and Post-Intervention.

### 3.4 Correlations between human milk bioactives depend on maternal diet and secretor status, an exploratory analysis

**Figure 2:**
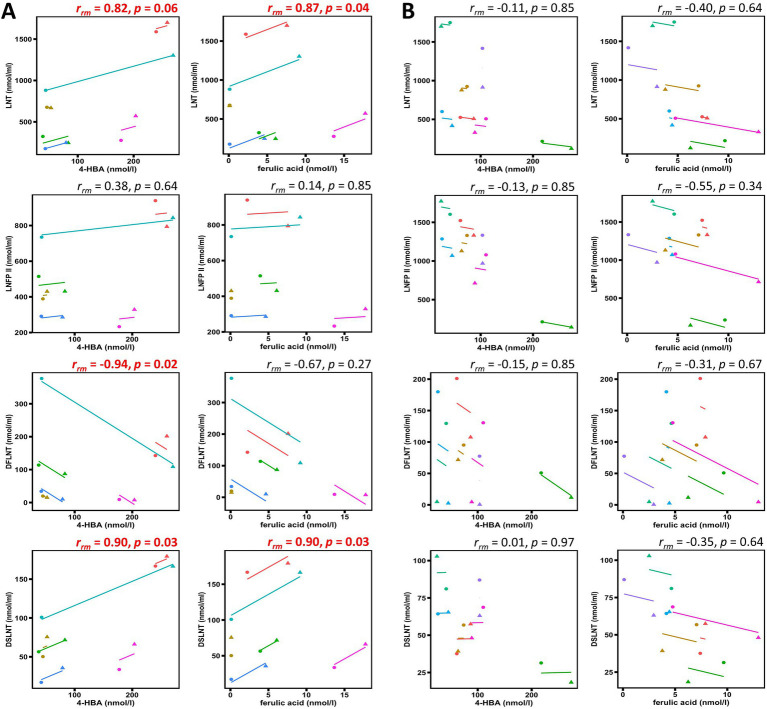
Scatterplots showing repeated measures correlations between human milk oligosaccharides and polyphenol metabolites. Correlation coefficients and *p*-values shown demonstrate within-participant associations amongst human milk bioactive components for **(A)** secretors and **(B)** non-secretors. Each color represents pre-intervention (●) and post-intervention (▲) measurements of different participants. The p-values shown are FDR-adjusted and correlations demonstrating a statistical trend (*p* < 0.1) or significance (*p* < 0.05) are highlighted in red. 4-HBA, 4-hydroxybenzoic acid; LNT, lacto-N-tetraose; LNFP II, lacto-N-fucopentaose II; DFLNT, difucosyllacto-N-tetraose; DSLNT, disialyllacto-N-tetraose.

An exploratory correlation analysis was carried out between 4-HBA and ferulic acid with LNT, LNFP II, DFLNT, and DSLNT (Figure 2) due to the overlap in maternal factors associated with profiles of these bioactives. For secretors, 4-HBA positively correlated with LNT (*r_rm_* = 0.82, *p* = 0.06) and DSLNT (*r_rm_* = 0.90, *p* = 0.03), and ferulic acid with LNT (*r_rm_* = 0.87, *p* = 0.04) and DSLNT (*r_rm_* = 0.90, *p* = 0.03). The only negative correlation for secretors was between 4-HBA and DFLNT (*r_rm_* = −0.94, *p* = 0.02). 4-HBA and ferulic acid were not significantly correlated with LNT, LNFP II, DFLNT, and DSLNT for non-secretors.

## 4 Discussion

There is growing evidence that exposures to bioactives such as carotenoids, polyphenols, and oligosaccharides from HM are associated with gut development and/or neurodevelopment in breastfed infants (9–11, 13, 17, 18, 46). Polyphenols are unique amongst these bioactives since broad exposures occur only through HM during the first 6 months of life due to low levels in infant formula (8). Substantial variability in composition of HM carotenoids, polyphenol metabolites, and oligosaccharides exists between women (5, 7, 17, 18, 25, 28), highlighting the need to understand the impact of maternal dietary and non-dietary factors on profiles of these HM bioactives. In this study, we explored relationships between maternal diet and secretor status on composition of HM carotenoids, polyphenol metabolites, and oligosaccharides. We report for the first time evidence to suggest that dietary modulation of select oligosaccharides and polyphenol metabolites in HM may be associated with maternal secretor status. We also observed that correlations between select polyphenol metabolites (4-HBA, ferulic acid) and HMOs (LNT, DFLNT, DSLNT) differed depending on maternal secretor status. These findings are suggestive of a link between oligosaccharide and polyphenol metabolite profiles in HM that is driven by both dietary and non-dietary factors.

### 4.1 Maternal diet and carotenoid profiles of human milk

Increasing maternal carotenoid intake through supplementation and single dietary components has been demonstrated to increase carotenoid concentrations in HM. (19–22) The present study expands on these findings by evaluating HM carotenoid concentrations within the context of a broad change in maternal dietary pattern consistent with the 2020 DGA (47) executed in a manner reflective of normal daily dietary intake compared to highly controlled dietary interventions commonly used. Although average daily intakes of β-cryptoxanthin, lutein+zeaxanthin, α-carotene, and β-carotene were consistently greater than average intakes for U.S. adults (45), we did not observe significant changes in HM carotenoid concentrations over the intervention timeframe. Previous reports have demonstrated that β-carotene supplementation (30 mg/d) (19) and dietary sources of carotenoids, such as orange sweet potatoes (12 mg β-carotene/d) and tangerines (5.3 mg β-cryptoxanthin/d) (22) increase concentrations of these carotenoids in HM after 3–4 weeks. In the present study, carotenoid intake was lower than these previous reports, with β-carotene intake reaching 12 mg on less than 50% of intake days across all participants during the intervention. Therefore, it is likely that daily fluctuations in carotenoid intake resulted in dietary intakes of these experimental patterns not being sufficient to elicit a more significant response in HM carotenoid concentrations. Establishing a direct link between maternal dietary patterns and HM carotenoid concentrations will require that future studies utilize a more targeted approach in designing meal plans with more consistent carotenoid levels within the DGA dietary patterns. Additionally, future studies utilizing these dietary patterns should take into consideration dietary factors, such as co-consumption of lipids, that can modify absorption of carotenoids (22, 48). It should also be noted that carotenoid concentrations decrease over time during lactation (6, 49), highlighting the need for randomized controlled trials (RCTs) with multiple longitudinal measurements to capture any modulation of these temporal changes.

### 4.2 Maternal diet-secretor status interaction and human milk bioactive composition

Appearance of polyphenol metabolites in HM occurs as a result of metabolism and absorption of dietary polyphenols. Various host-related factors are known to modify polyphenol bioavailability, including health status (50), gut microbiome composition (51), and genetics (52). Despite this, the only factor that has been identified to increase polyphenol concentrations in HM is maternal dietary polyphenol intake (7, 17, 18, 23). Previous reports have linked maternal dietary polyphenol intake derived from single dietary components to HM through pharmacokinetic studies (7, 23, 33) and short duration interventions (≤14 days) (17, 53). Maternal dietary polyphenol intake is associated with increased HM polyphenol concentrations, including isoflavones (53), urolithins (17), epicatechin metabolites (7), and quercetin (23). Our study expands on these findings by evaluating changes in HM polyphenol metabolite concentrations within the context of a broad change in maternal dietary patterns (i.e., multiple and varying sources of dietary polyphenols) that resulted in a 2X increase of polyphenol intake. In the present study, we demonstrated that this dietary pattern increases concentrations of the polyphenol metabolites 4-HBA, ferulic acid, and p-coumaric acid in HM by ~1.5X. In previous interventions, intake of 470–480 mg/d of polyphenols resulted in greater HM polyphenol concentrations (17, 18). Unlike dietary carotenoid intake, participants’ daily polyphenol intake was greater than 480 mg on >95% of intervention days. To our knowledge, this study is the first to show that a broad dietary change consistent with the 2020 DGA can modify polyphenol metabolite profiles of HM.

Previous reports demonstrate substantial variability in concentrations of polyphenols in HM, even in women consuming similar sources and amounts of dietary polyphenols (17, 18, 33). This study is the first to explore whether dietary modulation of HM polyphenol concentrations depends on maternal secretor status during a dietary intervention. Although HMO profiles depend on maternal secretor status (4, 54), there are no previous reports describing associations between secretor status and HM polyphenol metabolite profiles. In the present study, we demonstrate, for the first time, that higher dietary intake of polyphenols was differentially associated with 4-HBA and ferulic acid concentrations in HM depending on maternal secretor status. This suggests that secretor status may explain, at least in part, the variability in HM polyphenol profiles in women with similar polyphenol intake. Increased concentrations of 4-HBA and ferulic acid were observed for secretors, but not non-secretors, over the intervention time frame. The major dietary source of ferulic acid is whole grain intake (55), with >90% bound to non-starch polysaccharides (56), allowing release and absorption after microbial fermentation in the lower gastrointestinal tract (57). Appearance of 4-HBA in circulation, and subsequently in HM, occurs from microbial catabolism of anthocyanins (58, 59), other flavonoids (60), and phenolic acids including ferulic acid (61) and p-coumaric acid (58). Therefore, the diet-by-secretor status interaction that we observed for ferulic acid and 4-HBA suggests that secretor status may influence microbial polyphenol metabolism. Although maternal gut and milk microbiome composition was not evaluated in the present study, others have reported differences in both gut (62) and milk microbiome (34) composition between secretors and non-secretors. However, future studies will be needed to elucidate whether differences in gut or milk microbiome composition in secretors and non-secretors acts as a mediator between maternal dietary polyphenol intake and differential responses in HM polyphenol metabolite profiles.

Maternal secretor status is a well-established factor that drives differences in HMO profiles, with secretors having greater concentrations of α1-2 fucosylated HMOs (4, 63–65). There is also emerging clinical evidence demonstrating a direct link between maternal diet and HMO profiles (26, 27). In this study, we are demonstrating that the response of several HMOs (LNT, LNFP II, DFLNT, and DSLNT) to dietary modulation is differentially associated with maternal secretor status. Interestingly, the decrease in LNFP II and DFLNT, which our group identified in the original analysis (26), specifically occurred in non-secretors. We also observed a decrease in LNT and an increase in DSLNT in non-secretors and secretors, respectively. Our findings are consistent with a recent cross-sectional study suggesting that the correlation of maternal polyphenol intake with HMO profiles depends on maternal secretor status (25). Due to the focus on women with obesity and short duration of this pilot study, it will be important to include women of all weight statuses and longitudinal measurements over the course of lactation to elucidate factors driving the observed changes in HMO profiles.

To our knowledge, this pilot study represents the first example of a dataset that allows an integrated evaluation of interactions between maternal diet, secretor status, HMOs, and polyphenol metabolites in HM. The results of our correlation analysis suggest that relationships between oligosaccharides and polyphenols in HM may depend on maternal secretor status. We have shown that correlations between specific polyphenol metabolites (4-HBA, ferulic acid) and oligosaccharides (LNT, DFLNT, DSLNT) in HM were associated with maternal secretor status during a dietary intervention that increases polyphenol intake. Specifically, we observed that polyphenol metabolites and HMOs are significantly correlated in secretors, but not in non-secretors. This is suggestive of relationships between maternal secretor status and the dietary modulation of oligosaccharides and polyphenols in HM and is consistent with a previous report demonstrating that maternal polyphenol intake is a significant predictor of HMO profiles in secretors (25). Because a primary source of polyphenol metabolites in circulation, and subsequently in HM, is microbial metabolism of dietary polyphenols (35, 36), it is possible that the relationships between maternal diet, secretor status, and oligosaccharides and polyphenol metabolites in HM are mediated by the gut microbiota. Although we did not evaluate maternal gut microbiota in the present study, it has been demonstrated that intake of probiotics (66) modifies HMO profiles, suggesting a link between modulation of gut microbiome composition and HMO profiles. Additionally, dietary polyphenols have been associated with “prebiotic”-like effects leading to modulation of gut microbiome composition (67, 68). Therefore, future studies are warranted to explore whether polyphenol intake may modify HMO profiles via modulation of the gut microbiota as well as a combination of microbial and host metabolism. It is also possible that the interactions observed in the present study between maternal diet, secretor status, and HM bioactives may be mediated by HM microbiota. Interestingly, maternal secretor status (34) and diet (24) are associated with modulation of *Bifidobacterium* spp. in HM, with increases reported in secretors and with greater polyphenol intake. Certain species and strains of *Bifidobacterium*, some of which may be present in HM (69, 70), can assimilate complex fucosylated HMOs (71–73). It has been suggested that utilization of fucosylated HMOs is dependent on ATP-binding cassette (ABC) transporters (72), which can also transport polyphenols (74). The strategy for utilization of sialylated HMOs such as DSLNT by *Bifidobacterium* likely involves extracellular release of sialic acid (75), which may subsequently become available to other HM microbiota or for utilization to produce sialylated HMOs. Thus, the relationship between maternal secretor status, diet, and oligosaccharides and polyphenol metabolites in HM may reflect the interplay between available substrates and *Bifidobacterium* composition and abundance. However, future studies are needed to evaluate whether factors that modify *Bifidobacterium* composition and abundance (e.g., diet, secretor status) in HM influence the interplay between substrates such as HMOs, polyphenols, and monosaccharides derived from HMO breakdown.

### 4.3 Future research directions

The results of this study suggest that maternal FUT2 secretor status may be associated with differential responses in HM bioactive composition (HMOs, polyphenols) within the context of a healthy dietary pattern rich in polyphenols. Growing evidence suggests that these same dietary substrates may modulate the gut-brain axis in breastfed infants (9–12, 17, 76). However, several critical gaps exist limiting the translation of these results to developmental impacts for breastfed infants. Due to the complexity of factors likely driving profiles of HMOs and polyphenols, both longitudinal cohort and dietary intervention studies are required, and these studies should include a deeper phenotypic characterization of the mother-infant dyad throughout pregnancy and lactation. To understand potential interactions between maternal dietary and non-dietary factors on composition of HMOs and polyphenols, the following should be considered through application of systematic and integrated methodologies: (1) broader characterization of maternal diet composition, especially during critical stages of development such as lactation, (2) genetics (e.g., secretor status, phase II host metabolism enzymes), (3) health status (e.g., weight status, cardiometabolic biomarkers), and (4) gut microbiome composition. To evaluate the extent to which these HM bioactives may influence the gut-brain axis of breastfed infants, similar considerations are needed across infancy, childhood, and beyond, with the addition of the following: (1) quantification of breastfed infant intakes of both HMOs and individual polyphenols or metabolites through human milk and complimentary feeding, (2) omics data relevant to gut-brain axis development (e.g., short-chain fatty acids, gut microbiome, tryptophan metabolites), and (3) clinical outcomes relevant to cognitive development and function (e.g., cognitive assessment, EEG, MRI). Working to address these specific knowledge gaps will advance our understanding of human milk bioactives and their potential role in development of the gut-brain axis in breastfed infants.

### 4.4 Strengths and limitations

The results of this pilot study provide foundational information needed to design and conduct future studies exploring factors that influence HMO and polyphenol metabolite composition of HM and subsequent impacts on infant development. However, the study is limited by sample size highlighting a clear need to confirm the findings of our pilot study in a RCT, which will also be critical in identifying how dietary interventions can be leveraged for modifying composition of HM bioactives in a manner that promotes optimal infant development. A second limitation of the present study is the storage of HM samples during the 24-h collection period. The effect of participants storing HM samples at 4°C during this time period on stability of polyphenol metabolites was not investigated. A third limitation is the lack of a normal weight group since obesity is associated with modified HMO profiles (3) and polyphenol metabolism and absorption (50). There were several significant strengths to this study: (1) dietary modulation of multiple HM bioactives within the context of a dietary pattern consistent with the 2020 DGA recommendations (47); (2) simultaneous analyses of HMOs and polyphenol metabolites in HM, allowing for evaluation of associations between these two classes of HM bioactives for the first time; (3) inclusion of diet and a non-dietary factor (maternal secretor status) in an evaluation of polyphenol metabolites in HM; and (4) significant interactions between maternal diet and secretor status associated with differential profiles of HMOs and microbial-derived polyphenol metabolites in HM. Future studies need to incorporate a more integrated approach in RCTs involving a larger sample size that can encompass multiple non-dietary factors (e.g., weight status/BMI, secretor status, microbiome composition), longer intervention time frame, and ability to link maternal diet to changes in HM bioactive composition and infant developmental outcomes.

## 5 Conclusion

This study is the first to demonstrate that the impact of maternal diet on composition of HMOs and polyphenol metabolites in HM may depend on maternal secretor status. It is also the first to suggest a link between maternal diet, secretor status, HMOs, and microbial metabolites of dietary polyphenols. This investigation allows for a better understanding of factors driving HM bioactive composition and establishes a need for further studies, which could be leveraged for optimizing infant health and development.

## Data Availability

The original contributions presented in the study are included in the article/Supplementary material, further inquiries can be directed to the corresponding authors.
