# Supplementary material for: Dietary modulation of human milk bioactives is associated with maternal FUT2 secretor phenotype: an exploratory analysis of carotenoids and polyphenol metabolites
Source: Front Nutr. 2024 Oct 9;11:1463969. doi: 10.3389/fnut.2024.1463969 (PMC11496265; doi:10.3389/fnut.2024.1463969)
Supplement: Supplementary file 1 [file Table_1.docx]

| **Table S1.** UPLC-MS/MS parameters optimized for the characterization and quantitation of polyphenol metabolites in human milk.^a^ | | | | | | | | | |
| --- | --- | --- | --- | --- | --- | --- | --- | --- | --- |
| **Analyte** | **Alternative**  **Name** | **MW**  **(g/mol)** | **RT**  **(min)** | **Mode** | **[M-H]^b^**  **(*m/z*)** | **[M+H]^c^**  **(*m/z*)** | **Fragment**  **Ions (m/z)** | **CV (V)** | **CE**  **(eV)** |
| *Phenolic acids* |  |  |  |  |  |  |  |  |  |
| 3,4-dihydroxybenzaldehyde |  | 138.12 | 1.84 | ES+ |  | 138.90 | 111.00, **65.01** | 10 | 8, 22 |
| 4-hydroxybenzoic acid |  | 138.12 | 1.43 | ES+ |  | 139.00 | 121.00, **95.00** | 30 | 12 |
| 3,4-dihydroxybenzoic acid | protocatechuic acid | 154.12 | 1.43 | ES+ |  | 155.00 | 93.33, **64.97** | 28 | 25 |
| 4-hydroxycinnamic acid | *p*-coumaric acid | 164.16 | 3.30 | ES+ |  | 165.10 | 147.00, **119.00** | 30 | 20 |
| 3,4-dihydroxycinnamic acid | caffeic acid | 180.16 | 2.60 | ES+ |  | 181.00 | 163.00, **145.00** | 30 | 20 |
| 4-hydroxy-3-methoxycinnamic acid | ferulic acid | 194.18 | 3.94 | ES+ |  | 194.90 | **145.00**, 117.00 | 18 | 16, 20 |
| 3,4-dihydroxyphenylacetic acid |  | 168.15 | 1.75 | ES- | 166.90 |  | **123.00**, 121.00 | 10 | 10 |
| 3,4,5-trihydroxybenzoic acid | gallic acid | 170.12 | 0.80 | ES- | 168.83 |  | 97.10, **78.88** | 34, 36 | 16, 22 |
| 3-(3,4-dihydroxyphenyl)propionic acid | dihydrocaffeic acid | 182.17 | 2.32 | ES- | 180.82 |  | 108.84, **58.82** | 28 | 12, 14 |
| 4-hydroxyhippuric acid |  | 195.17 | 1.58 | ES- | 194.00 |  | 121.00, **93.00** | 32 | 24, 22 |
| 3-(4-hydroxy-3-methoxyphenyl)propionic acid | dihydroferulic acid | 196.20 | 3.77 | ES- | 194.85 |  | **135.83**, 120.88 | 32 | 12, 24 |
| dihydrocaffeic acid 3-*O*-sulfate |  | 262.24 | 2.67 | ES- | 261.00 |  | **181.00**, 137.00 | 10 | 13, 34 |
| ferulic acid 4-*O*-sulfate |  | 274.25 | 3.70 | ES- | 273.00 |  | **193.00**, 134.00 | 30 | 12, 18 |
| benzoic acid 4-*O*-glucuronide |  | 314.24 | 1.17 | ES- | 312.89 |  | **112.86**, 92.96 | 20 | 10, 34 |
| 4-*O*-caffeoylquinic acid | cryptochlorogenic acid | 354.31 | 1.82 | ES- | 353.02 |  | 190.97, **85.00** | 26 | 18, 40 |
| 5-*O*-caffeoylquinic acid | neochlorogenic acid | 354.31 | 2.37 | ES- | 353.02 |  | 190.97, **85.00** | 26 | 18, 40 |
| 3-*O*-caffeoylquinic acid | chlorogenic acid | 354.31 | 2.61 | ES- | 353.02 |  | 190.97, **85.00** | 26 | 18, 40 |
| caffeic acid 4-*O*-glucuronide |  | 356.28 | 1.80 | ES- | 354.99 |  | 179.00, **134.90** | 30, 10 | 18, 40 |
| caffeic acid 3-*O*-glucuronide |  | 356.28 | 2.37 | ES- | 354.99 |  | 179.00, **134.90** | 30, 10 | 18, 40 |
| ferulic acid 4-*O*-glucuronide |  | 370.31 | 2.17 | ES- | 369.00 |  | **193.00**, 178.10 | 30, 10 | 22, 32 |
| dihydroferulic acid 4-*O*-glucuronide |  | 372.32 | 2.75 | ES- | 371.00 |  | **195.00**, 120.90 | 10 | 22, 34 |
| *Flavonoids* |  |  |  |  |  |  |  |  |  |
| naringenin |  | 272.25 | 5.39 | ES- | 271.03 |  | 150.96, **118.98** | 40 | 18, 26 |
| kaempferol |  | 286.24 | 5.48 | ES- | 285.02 |  | **150.97**, 145.63 | 58 | 18, 30 |
| catechin |  | 290.27 | 2.22 | ES- | 289.10 |  | 123.00, **109.00** | 33 | 26, 24 |
| epicatechin |  | 290.27 | 3.08 | ES- | 289.10 |  | 123.00, **109.00** | 33 | 26, 24 |
| gallocatechin |  | 306.27 | 1.48 | ES- | 304.98 |  | 178.98, **124.95** | 40 | 14, 20 |
| epigallocatechin |  | 306.27 | 2.05 | ES- | 304.98 |  | 178.98, **124.95** | 40 | 14, 20 |
| myricetin |  | 318.24 | 4.69 | ES- | 317.02 |  | 178.95, **150.99** | 46 | 20, 26 |
| epigallocatechin gallate |  | 458.37 | 3.15 | ES- | 457.02 |  | **168.96**, 124.94 | 30 | 16, 40 |
| hesperetin 3-*O*-glucuronide |  | 478.40 | 4.96 | ES- | 476.92 |  | 301.00, **112.93** | 2 | 24, 18 |
| *Urolithins* |  |  |  |  |  |  |  |  |  |
| isourolithin A |  | 228.20 | 4.91 | ES+ |  | 228.85 | **156.96**, 127.77 | 40 | 20, 30 |
| urolithin A |  | 228.20 | 4.95 | ES+ |  | 228.85 | **156.96**, 127.77 | 40 | 20, 30 |
| isourolithin A 9-glucuronide |  | 404.30 | 3.92 | ES- | 403.00 |  | **227.00**, 198.00 | 10 | 34, 42 |
| *Stilbenes* |  |  |  |  |  |  |  |  |  |
| dihydroresveratrol |  | 230.26 | 4.93 | ES- | 229.09 |  | 143.00, **122.04** | 38 | 20, 26 |
| resveratrol 3-*O*-sulfate |  | 308.31 | 4.78 | ES- | 306.90 |  | **227.03**, 40.87 | 10 | 20, 38 |
| *Internal standards* |  |  |  |  |  |  |  |  |  |
| Taxifolin |  | 304.25 | 4.10 | ES- | 303.00 |  | 177.00, **125.00** | 34 | 14, 22 |
| Ethyl gallate |  | 198.17 | 3.51 | ES- | 197.00 |  | 125.00, **124.00** | 30 | 30 |
| ^a^Fragment ions in bold were used for quantitation. MW: molecular weight; RT: retention time; CV: cone voltage; CE: collision energy.  ^b^Negative mode (ES-) conditions: desolvation temperature 600 ℃, desolvation gas flow 800 L h^-1^, capillary voltage -2.5 kV, cone voltage 32 V, and collision energy 3 V  ^c^Positive mode (ES+) conditions: desolvation temperature 600 ℃, desolvation gas flow 800 L h^-1^, capillary voltage 3 kV, cone voltage 30 V, and collision energy 12 V | | | | | | | | | |

| **Table S2.** Validation parameters for polyphenol metabolites extracted from human milk. | | | | | | | | | |
| --- | --- | --- | --- | --- | --- | --- | --- | --- | --- |
| **Analyte** | **Alternative name** | **Equation** | **R^2^** | **Range (nM)** | **LOD (nM)** | **LOQ (nM)** | **Recovery%**  **(mean ± SD)** | **CV** | **ME** |
| 3,4-dihydroxybenzaldehyde | protocatechuicaldehyde | y=45.66x+18.13 | 0.9980 | 2.44-156.25 | 2.04 | 6.18 | 92.09 ± 0.61 | 0.66% | 0.98 |
| 4-hydroxybenzoic acid |  | y=199.04x + 291.75 | 0.9994 | 1.22-78.13 | 1.31 | 3.96 | 104.88 ± 1.02 | 0.97% | 0.59 |
| 3,4-dihydroxybenzoic acid | protocatechuic acid | y=37.43x-15.02 | 0.9939 | 2.44-156.25 | 3.50 | 10.60 | 100.01 ± 0.65 | 0.65% | 0.48 |
| 4-hydroxycinnamic acid | *p*-coumaric acid | y=300.21x-35.04 | 0.9997 | 0.61-39.06 | 0.29 | 0.87 | 82.29 ± 0.89 | 1.08% | 0.92 |
| 3,4-dihydroxycinnamic acid | caffeic acid | y=63.80x+40.51 | 0.9987 | 2.44-156.25 | 3.16 | 9.56 | 87.11 ± 0.97 | 1.11% | 0.82 |
| 4-hydroxy-3-methoxycinnamic acid | ferulic acid | y=306.26x+16.27 | 0.9995 | 0.61-39.06 | 0.56 | 1.71 | 72.10 ± 1.24 | 1.72% | 0.97 |
| isourolithin A |  | y=286.85x-0.41 | 0.9994 | 0.61-39.06 | 0.47 | 1.41 | 39.22 ± 1.27 | 3.24% | 1.09 |
| urolithin A |  | y=568.00x-34.41 | 0.9998 | 0.61-39.06 | 0.30 | 0.91 | 36.62 ± 1.49 | 4.07% | 0.84 |
| 3,4-dihydroxyphenylacetic acid |  | y=3.99x-64.14 | 0.9998 | 39.06-2,500 | 32.57 | 98.69 | 83.63 ± 0.47 | 0.56% | 0.95 |
| 3,4,5-trihydroxybenzoic acid | gallic acid | y=4.72x-63.90 | 0.9996 | 39.06-2,500 | 45.78 | 138.72 | 106.21 ± 3.40 | 3.20% | 0.51 |
| 3-(3,4-dihydroxyphenyl)propionic acid | dihydrocaffeic acid | y=30.06x-5.34 | 0.9992 | 0.61-78.13 | 1.59 | 4.81 | 92.03 ± 1.81 | 1.97% | 0.79 |
| 4-hydroxyhippuric acid |  | y=23.31x-6.86 | 0.9983 | 1.22-156.25 | 0.56 | 1.69 | 91.22 ± 1.04 | 1.14% | 0.83 |
| 3-(4-hydroxy-3-methoxyphenyl)propionic acid | dihydroferulic acid | y=7.74x-82.03 | 0.9979 | 9.77-625 | 7.90 | 23.90 | 82.29 ± 1.30 | 1.58% | 1.04 |
| dihydroresveratrol |  | y=0.83x+25.44 | 0.9917 | 156.25-2,500 | 224.40 | 680.01 | 74.42 ± 6.33 | 8.51% | 0.84 |
| dihydrocaffeic acid 3-*O*-sulfate |  | y=233.01x-105.51 | 0.9971 | 0.61-39.06 | 0.70 | 2.12 | 79.25 ± 1.33 | 1.68% | 1.24 |
| naringenin |  | y=76.13x+85.01 | 0.9903 | 0.31-39.06 | 0.88 | 2.66 | 18.40 ± 1.10 | 5.98% | 0.82 |
| ferulic acid 4-*O*-sulfate |  | y=129.58x-106.99 | 0.9981 | 0.61-39.06 | 0.43 | 1.30 | 73.15 ± 1.28 | 1.75% | 1.32 |
| kaempferol |  | y=2.08x-82.61 | 0.9995 | 78.13-5,000 | 103.68 | 314.19 | 2.01 ± 0.42 | 20.90% | 1.03 |
| catechin |  | y=12.15x-69.45 | 0.9983 | 9.78-625 | 12.31 | 37.29 | 78.13 ± 1.15 | 1.47% | 1.06 |
| epicatechin |  | y=12.84x-64.82 | 0.9997 | 19.53-1,250 | 16.90 | 51.23 | 92.75 ± 1.45 | 1.56% | 0.99 |
| gallocatechin |  | y=30.65x-5.98 | 0.9995 | 1.22-156.25 | 1.00 | 3.03 | 79.58 ± 0.62 | 0.78% | 1.00 |
| epigallocatechin |  | y=39.39x-160.48 | 0.9989 | 4.88-312.5 | 5.47 | 16.57 | 82.55 ± 0.45 | 0.55% | 1.06 |
| resveratrol 3-*O*-sulfate |  | y=428.75x-301.01 | 0.9950 | 0.61-39.06 | 0.41 | 1.25 | 9.88 ± 0.94 | 9.51% | 0.83 |
| benzoic acid 4-*O*-glucuronide |  | y=28.71x-6.66 | 0.9957 | 1.22-78.13 | 1.43 | 4.35 | 80.25 ± 0.98 | 1.22% | 0.93 |
| myricetin |  | y=27.72x-65.89 | 0.9993 | 4.88-312.5 | 5.13 | 15.55 | 3.59 ± 0.71 | 19.78% | 1.18 |
| 4-*O*-caffeoylquinic acid | cryptochlorogenic acid | y=6.38x-25.40 | 0.9972 | 9.77-625 | 13.34 | 40.42 | 87.70 ± 1.67 | 1.90% | 1.01 |
| 5-*O*-caffeoylquinic acid | neochlorogenic acid | y=9.33x-22.45 | 0.9983 | 4.88-312.5 | 6.19 | 18.75 | 91.90 ± 1.16 | 1.26% | 1.04 |
| 3-*O*-caffeoylquinic acid | chlorogenic acid | y=4.18x-16.28 | 0.9991 | 4.88-312.5 | 3.66 | 11.09 | 90.82 ± 2.25 | 2.48% | 0.92 |
| caffeic acid 4-*O*-glucuronide |  | y=57.16x-21.73 | 0.9978 | 0.61-39.06 | 0.80 | 2.41 | 81.25 ± 0.70 | 0.86% | 0.97 |
| caffeic acid 3-*O*-glucuronide |  | y=113.34x+129.05 | 0.9991 | 2.44-156.25 | 3.54 | 10.73 | 64.04 ± 0.99 | 1.18% | 1.07 |
| ferulic acid 4-*O*-glucuronide |  | y=24.87x-19.50 | 0.9994 | 2.44-156.25 | 2.14 | 6.48 | 88.46 ± 1.07 | 1.21% | 0.99 |
| dihydroferulic acid 4-*O*-glucuronide |  | y=20.48x-84.27 | 0.9992 | 9.77-625 | 11.96 | 36.26 | 92.93 ± 1.92 | 2.07% | 0.96 |
| isourolithin A 9-glucuronide |  | y=47.73x-33.68 | 0.9992 | 1.22-156.25 | 2.67 | 8.08 | 67.12 ± 1.74 | 2.59% | 0.91 |
| epigallocatechin gallate |  | y=27.22x-141.55 | 0.9976 | 2.44-312.5 | 1.99 | 6.03 | 0.11 ± 0.04 | 36.36% | 1.08 |
| hesperetin 3-*O*-glucuronide |  | y=43.95x-227.50 | 0.9963 | 4.88-312.5 | 3.25 | 9.85 | 77.54 ± 1.21 | 1.56% | 1.03 |
| Abbreviations are as follows: LOD = limit of detection; LOQ = limit of quantitation; CV = coefficient of variation; ME = matrix effect | | | | | | | | | |

| **Table S3.** Interquartile range (IQR) for daily phytochemical intake over the 4-week dietary intervention | |
| --- | --- |
| **Carotenoids** | **IQR** |
| *β-cryptoxanthin (μg/d)* | 47-367 |
| *lutein+zeaxanthin (μg/d)* | 2,972-7,591 |
| *β-carotene (μg/d)* | 2,767-30,342 |
| *α-carotene (μg/d)* | 455-1,054 |
| *lycopene (μg/d)* | 0-2,045 |
| **Polyphenols** |  |
| *total polyphenols (mg/d)* | 1,249-2,642 |

| Table S4. Results from non-parametric 2-way repeated measures ANOVA models for human milk bioactives and sensitivity analysis of α-carotene and β-carotene | | | | |
| --- | --- | --- | --- | --- |
|  | *p-value of variable* | | | |
| bioactive component | **all participants** | **secretors** | **non-secretors** | **interaction term**  **(time*secretor status)** |
| *CAROTENOIDS* |  |  |  |  |
| lutein | 0.16 | 0.75 | **0.06** | 0.36 |
| zeaxanthin | 0.29 | 0.43 | 0.46 | 0.94 |
| β-cryptoxanthin | 0.49 | 1.00 | **0.09** | 0.49 |
| α-carotene | 0.11 | 0.13 | 0.59 | 0.30 |
| β-carotene | 0.22 | 0.49 | 0.25 | 0.94 |
| lycopene | 0.40 | 0.95 | 0.13 | 0.45 |
| *POLYPHENOL METABOLITES* |  |  |  |  |
| 4-hydroxybenzoic acid | **0.01** | **0.02** | 0.37 | **0.08** |
| *p*-coumaric acid | **0.02** | 0.25 | **0.03** | 0.51 |
| ferulic acid | **0.05** | **0.006** | 0.78 | **0.02** |
| gallic acid | 0.17 | 0.11 | 0.45 | 0.99 |
| 4-hydroxyhippuric acid | 0.69 | 0.42 | 0.65 | 0.35 |
| dihydroferulic acid | 0.20 | 0.74 | **0.02** | 0.50 |
| myricetin | 0.64 | 0.94 | 0.61 | 0.71 |
| caffeic acid 4-*O*-glucuronide | 0.48 | 0.34 | 0.93 | 0.55 |
| hesperetin 3-*O*-glucuronide | 0.56 | 0.84 | 0.17 | 0.36 |
| *OLIGOSACCHARIDES* |  |  |  |  |
| 2'-fucosyllactose (2'FL) | 0.47 | 0.22 | 0.70 | 1.00 |
| 3-fucosyllactose (3FL) | 0.74 | 0.29 | 0.68 | 0.33 |
| 3'-sialyllactose (3'SL) | 0.63 | 0.34 | 0.84 | 0.93 |
| difucosyllactose (DFLac) | 0.41 | 1.00 | 0.26 | 0.41 |
| 6'-sialyllactose (6'SL) | **0.09** | 0.25 | 0.21 | 0.91 |
| lacto-N-tetraose (LNT) | 0.62 | 0.24 | **0.000004** | **0.007** |
| lacto-N-neotetraose (LNnT) | 0.59 | 0.79 | 0.39 | 0.39 |
| lacto-N-fucopentaose I (LNFP I) | 0.40 | 0.87 | 0.17 | 0.59 |
| lacto-N-fucopentaose II (LNFP II) | **0.05** | 0.68 | **0.008** | **0.02** |
| lacto-N-fucopentaose III (LNFP III) | **0.009** | 0.48 | **0.002** | 0.12 |
| sialyllacto-N-tetraose b (LSTb) | 0.70 | 0.57 | 0.27 | 0.24 |
| sialyllacto-N-tetraose c (LSTc) | 0.71 | 0.77 | 0.49 | 0.47 |
| difucosyllacto-N-tetraose (DFLNT) | **0.000004** | 0.10 | **0.00001** | **0.003** |
| lacto-N-hexaose (LNH) | 0.99 | 0.77 | 0.81 | 0.71 |
| disialyllacto-N-tetraose (DSLNT) | 0.11 | **0.0008** | 0.42 | **0.005** |
| fucosyllacto-N-hexaose (FLNH) | 0.63 | 0.41 | 0.17 | 0.11 |
| difucosyllacto-N-hexaose (DFLNH) | 0.78 | 0.94 | 0.78 | 0.85 |
| disialyllacto-N-hexaose (DSLNH) | **0.08** | 0.12 | 0.29 | 0.96 |
|  |  |  |  |  |
| sensitivity analysis |  |  |  |  |
| α-carotene | 0.19 | 0.24 | 0.55 | 0.49 |
| β-carotene | 0.20 | 0.49 | 0.18 | 0.89 |
| ANOVA model: carotenoid ~ time * secretor status.  P-values for all participants derived from the time term (Pre vs. Wk4) in the model. P-values for secretors and non-secretors derived from post-hoc analyses. Non-parametric ANOVA models carried out using nparLD R package which estimates ANOVA models after rank-based transformation. P-values demonstrating a statistical trend (P < 0.1) or significance (P < 0.05) are highlighted in red. | | | | |
